# Lymph node stromal topology as a hidden regulator of breast cancer outcome: commentary on reticular network analysis^†^

**DOI:** 10.1002/path.70085

**Published:** 2026-05-27

**Authors:** Andrea Vethencourt, Roberto Salgado, Eva Gonzalez‐Suarez

**Affiliations:** ^1^ Institut Català d'Oncologia, Medical Oncology Department Breast Cancer Unit Barcelona Spain; ^2^ IDIBELL, Institut d'Investigació Biomèdica de Bellvitge Barcelona Spain; ^3^ Department of Pathology ZAS Hospitals Antwerp Belgium; ^4^ Division of Research Peter MacCallum Cancer Centre Melbourne Victoria Australia; ^5^ Centro Nacional de Investigaciones Oncológicas (CNIO) Madrid Spain

**Keywords:** breast cancer, lymph node, stromal topology, fibroblastic reticular cells, spatial biomarkers

## Abstract

The axillary lymph node remains a cornerstone of breast cancer staging and therapeutic decision‐making, yet it is still largely interpreted through a static anatomical framework. Emerging evidence challenges this paradigm, positioning tumor‐draining lymph nodes as dynamic immunological ecosystems that actively regulate tumor progression and host response. In this context, Llewellyn *et al* introduce a quantitative framework to characterize fibroblastic reticular cell network topology and link nodal architecture to clinical outcomes. By quantifying features such as lacunarity, branching, and alignment in a breast cancer cohort, the authors show that fibroblastic reticular cell network organization is associated with prognosis in a context‐dependent manner. While this approach represents a significant conceptual and methodological advance, its biological interpretation remains limited by the absence of functional validation and integrated immune profiling. Accumulating evidence indicates that lymph node microenvironments undergo profound immune reprogramming, highlighting the close interdependence between stromal topology and immune composition. These findings suggest that structural features alone may reflect distinct underlying biological states. Integrating spatial metrics with immune characterization may refine current staging systems, improve risk stratification, and ultimately inform future evolution of staging classifications by incorporating functional and spatial dimensions of nodal involvement. © 2026 The Author(s). *The Journal of Pathology* published by John Wiley & Sons Ltd on behalf of The Pathological Society of Great Britain and Ireland.

The axillary lymph node remains a central determinant in breast cancer management, serving as a cornerstone of staging systems and a key determinant of therapeutic decision‐making [1, 2]. Nodal status remains one of the most powerful prognostic factors, guiding both systemic treatment strategies and the extent of axillary surgery [3, 4, 5, 6]. However, this paradigm is largely grounded in a static and anatomical interpretation of the lymph node, viewed primarily as a passive reservoir of metastatic cells. Such a framework risks overlooking the complex immunological and stromal interactions within the nodal microenvironment that may critically influence tumor progression and host response.

Biologically, axillary lymph nodes perform a dual function: they serve as sites for antigen presentation and adaptive immune activation, while also representing the first anatomical station for metastatic dissemination [7]. Tumor cells are known to evade immune surveillance within lymph nodes by actively remodeling their microenvironment, for example through the induction of lymphangiogenesis and stromal reprogramming [8]. Increasing evidence suggests that lymph nodes should therefore be regarded not merely as indicators of disease spread, but as dynamic immunological ecosystems whose structural organization may shape both anti‐tumor immunity and metastatic potential. In this context, the study by Llewellyn *et al* provides important new insights by analyzing the topology of the fibroblastic reticular cell (FRC) network in human axillary lymph nodes and linking its spatial organization to clinical outcomes in breast cancer [9]. This approach moves beyond descriptive histopathology toward quantifiable architecture, highlighting a previously underexplored layer of biological information embedded within the nodal architecture.

From a biological perspective, the authors propose an appealing mechanistic framework, in which the organization of the FRC network modulates lymphatic flow, antigen distribution, and immune cell interactions within the node. This raises the possibility that stromal topology does not merely reflect disease status but actively participates in regulating immune competence and metastatic progression. However, these interpretations remain largely inferential, as the study is based on static morphological analyses, and lacks direct functional validation.

Future work integrating dynamic models to interrogate cellular trafficking and immune activation will be important to establish causality and define the biological significance of these architectural changes. The quantitative characterization of stromal topology proposed by Llewellyn *et al* represents a clinically relevant advance, providing measurable features of lymph node architecture with prognostic potential [9]. Integrating these spatial metrics with high‐dimensional immune profiling would further enhance their interpretative value. Supporting this, recent studies show that fibroblastic reticular cells actively regulate immune cell recruitment and function, while single‐cell analyses reveal profound immune reprogramming within lymph nodes, including reduced T cell activation and impaired antigen presentation [10, 11, 12]. Together, these data indicate that stromal architecture and immune composition are tightly linked. While topological features are intrinsically informative, their full biological and clinical significance will be best realized when interpreted alongside the immune context in which they arise.

One of the most intriguing aspects of the study is the context‐dependent interpretation of key topological parameters, particularly lacunarity. In treatment‐naïve triple negative breast cancer, uninvolved lymph nodes exhibited a more compact and highly branched FRC network, characterized by lower lacunarity and increased branchpoints, compared with reactive nodes. Interestingly, neoadjuvant chemotherapy induced similar patterns of network compaction across breast cancer subtypes; however, the prognostic implications of these changes appeared to diverge according to clinical context. This apparent discordance highlights the complexity of interpreting stromal architecture in isolation and suggests that identical structural features may reflect fundamentally different biological states.

One possible explanation is that more aggressive tumors may ‘precondition’ the lymph node microenvironment even in the absence of overt metastasis, through the release of soluble factors or extracellular vesicles that drive early stromal remodeling. In this scenario, a more compact FRC network could represent distinct functional states, potentially enhancing treatment responsiveness in certain contexts, while in others promoting immune evasion or facilitating tumor progression. These findings reinforce the concept that lymph node architecture is dynamically shaped by tumor‐derived signals and underscore the need to interpret spatial biomarkers within their specific biological and clinical context.

Methodologically, the study represents a significant advance by introducing a quantitative, image‐based framework to capture the spatial complexity of the lymph node microenvironment. In particular, the use of the computational pipeline TWOMBLI enables systematic quantification of key topological features of the FRC network, including lacunarity (reflecting the size and distribution of network voids), branching, network density, and fiber alignment. By transforming histological architecture into measurable and reproducible parameters, this approach moves beyond qualitative assessment and establishes a more objective basis for interrogating stromal organization. More broadly, the integration of such computational tools into tissue analysis marks an important step toward scalable, standardized, and potentially clinically translatable, but it remains unclear whether stromal topology adds independent predictive value beyond established biomarkers.

From a clinical standpoint, these results are provocative and raise important questions for the future of breast cancer staging and risk stratification. Should lymph nodes be interpreted solely based on the presence or absence of metastasis, or should we begin to incorporate functional and biological characteristics of the nodal microenvironment? Does the current TNM classification sufficiently capture the complexity of nodal involvement, or could it be refined by integrating spatial and stromal biomarkers? While the immediate clinical applicability of these findings remains uncertain, due to cohort heterogeneity, lack of standardization, and absence of prospective validation, the study provides a compelling proof of concept. It suggests that incorporating unbiased computational analyses of lymph node architecture may enhance patient stratification and open new avenues for understanding how tumors and therapies interact with the immune system.

The relevance of these findings is particularly apparent when considered alongside ongoing efforts to de‐escalate axillary surgery, such as the TADPOLE trial, which is evaluating targeted axillary dissection versus axillary clearance in patients with node‐positive early breast cancer [13]. This, and other trials, are driven by a fundamental clinical question: how much information is required from the axilla to guide treatment without compromising outcomes? In this context, the work by Llewellyn *et al* [9] introduces a complementary dimension, suggesting that lymph nodes may provide not only binary information on metastatic involvement, but also quantitative and biologically meaningful data embedded within their stromal architecture. Integrating such spatially resolved metrics could, in the future, refine patient selection. While these concepts remain exploratory, they point toward a model in which nodal assessment evolves from purely anatomical staging toward a more functional and biologically informed framework, with potential implications for both surgical decision‐making and systemic therapy selection.

The work by Llewellyn *et al* [9] also represents a meaningful conceptual advance. It challenges the traditional view of the lymph node as a passive indicator of disease burden and instead positions it as an active, dynamic regulator of tumor progression and immune competence. The clinical relevance of this nodal ecosystem is increasingly supported by emerging evidence suggesting that preservation of tumor‐draining lymph nodes may be critical for optimal therapeutic efficacy [14, 15]. These observations underscore the need to better understand how therapeutic interventions reshape the lymph node microenvironment. It will be particularly important for future studies by Llewellyn and colleagues to validate these findings in more contemporary cohorts and to determine whether the stromal alterations observed following neoadjuvant chemotherapy are maintained or modified when chemotherapy is combined with immune checkpoint inhibitors, which are now part of the standard of care in early triple‐negative breast cancer [16, 17].

Fully realizing the clinical potential of this paradigm will require rigorous validation and functional integration. Nevertheless, the direction is clear: incorporating spatial biology of the lymph node into our conceptual and clinical frameworks may fundamentally redefine how we interpret disease progression, moving beyond the view of lymph nodes as mere reservoirs of metastasis toward recognizing their full role as active regulators of tumor immune interactions.

## Author contributions statement

AV conceived the work and wrote the manuscript. RS and EG‐S contributed to critical revision of the manuscript for important intellectual content, provided expert analysis, and approved the final version.

## Data Availability

Data sharing not applicable to this article as no datasets were generated or analyzed during the current study.

## References

[1] Amin MB , Greene FL , Edge SB , *et al*. The eighth edition AJCC cancer staging manual: continuing to build a bridge from a population‐based to a more “personalized” approach to cancer staging. CA Cancer J Clin 2017; 67: 93–99. 28094848 10.3322/caac.21388

[2] Breast Cancer ‐ Guidelines Detail [Internet]. [cited 2026 Mar 30], Available from: https://www.nccn.org/guidelines/guidelines-detail?id=1419.

[3] Bartels SAL , Donker M , Poncet C , *et al*. Radiotherapy or surgery of the axilla after a positive sentinel node in breast cancer: 10‐year results of the randomized controlled EORTC 10981‐22023 AMAROS trial. J Clin Oncol 2023; 41: 2159–2165. 36383926 10.1200/JCO.22.01565

[4] Giuliano AE , Ballman KV , McCall L , *et al*. Effect of axillary dissection vs. no axillary dissection on 10‐year overall survival among women with invasive breast cancer and sentinel node metastasis: the ACOSOG Z0011 (alliance) randomized clinical trial. JAMA 2017; 318: 918–926. 28898379 10.1001/jama.2017.11470 PMC5672806

[5] Reimer T , Stachs A , Veselinovic K , *et al*. Axillary surgery in breast cancer — primary results of the INSEMA trial. N Engl J Med 2025; 392: 1051–1064. 39665649 10.1056/NEJMoa2412063

[6] Bromham N , Schmidt‐Hansen M , Astin M , *et al*. Axillary treatment for operable primary breast cancer. Cochrane Database Syst Rev 2017; 1: CD004561. 28052186 10.1002/14651858.CD004561.pub3 PMC6464919

[7] Jana S , Muscarella RA , Jones D . The multifaceted effects of breast cancer on tumor‐draining lymph nodes. Am J Pathol 2021; 191: 1353–1363. 34043978 10.1016/j.ajpath.2021.05.006 PMC8351123

[8] Eichin D , Lehotina D , Kauko A , *et al*. Breast cancer remodels lymphatics in sentinel lymph nodes. Nat Commun 2025; 16: 10056. 41249124 10.1038/s41467-025-64981-z PMC12623973

[9] Llewellyn AM , D'Costa SL , Lam CYR , *et al*. Topological analysis of the human lymph node reticular network predicts outcome in breast cancer. J Pathol 2026; 269: 348–362. 42003567 10.1002/path.70065 PMC13238348

[10] Mattavelli G , Helal M , Cetkovic A , *et al*. A TLR4‐dependent fibroblast‐monocyte axis in tumor‐draining lymph nodes contributes to metastasis in triple‐negative breast cancer. Immunity 2025; 58: 2830–2846.e15. 40957419 10.1016/j.immuni.2025.08.015

[11] Liu T , Liu C , Yan M , *et al*. Single cell profiling of primary and paired metastatic lymph node tumors in breast cancer patients. Nat Commun 2022; 131: 6823. 10.1038/s41467-022-34581-2 PMC9649678 36357424

[12] Guo W , Tan J , Wang L , *et al*. Tumor draining lymph nodes connected to cold triple‐negative breast cancers are characterized by Th2‐associated microenvironment. Nat Commun 2024; 151: 8592. 10.1038/s41467-024-52577-y PMC11452381 39366933

[13] TADPOLE|Association of Breast Surgery [Internet]. [cited 2026 Apr 27], Available from: https://associationofbreastsurgery.org.uk/professionals/research/trials/open-recruiting/tadpole.

[14] Koukourakis MI , Giatromanolaki A . Tumor draining lymph nodes, immune response, and radiotherapy: towards a revisal of therapeutic principles. Biochim Biophys Acta Rev Cancer 2022; 1877: 188704. 35227831 10.1016/j.bbcan.2022.188704

[15] Xu Q , Chu J , Hu Q , *et al*. The role and clinical significance of tumor‐draining lymph nodes in tumor progression and immunotherapy. Crit Rev Oncol Hematol 2025; 212: 104745. 40315968 10.1016/j.critrevonc.2025.104745

[16] Loibl S , André F , Bachelot T , *et al*. Early breast cancer: ESMO clinical practice guideline for diagnosis, treatment and follow‐up. Ann Oncol 2024; 35: 159–182. 38101773 10.1016/j.annonc.2023.11.016

[17] Schmid P , Cortes J , Dent R , *et al*. Overall survival with pembrolizumab in early‐stage triple‐negative breast cancer. N Engl J Med 2024; 391: 1981–1991. 39282906 10.1056/NEJMoa2409932

